# Drugs use pattern for uncomplicated malaria in medicine retail outlets in Enugu urban, southeast Nigeria: implications for malaria treatment policy

**DOI:** 10.1186/1475-2875-13-243

**Published:** 2014-06-24

**Authors:** Charles C Ezenduka, Brian O Ogbonna, Obinna I Ekwunife, Mathew J Okonta, Charles O Esimone

**Affiliations:** 1 Department of Clinical Pharmacy and Pharmacy Management, Faculty of Pharmaceutical Sciences, Nnamdi Azikiwe University, Awka, Nigeria; 2 Department of Clinical Pharmacy and Pharmacy Management, Faculty of Pharmaceutical Sciences, University of Nigeria, Nsukka, Nigeria; 3 Department of Pharmaceutical Microbiology and Biotechnology, Faculty of Pharmaceutical Sciences, Nnamdi Azikiwe University, Awka, Nigeria

**Keywords:** Anti-malarial drugs, Utilization pattern, Artemisinin-based combination therapy, Private retail sector, Affordable Medicines Facility-malaria

## Abstract

**Background:**

Malaria treatment policy recommends regular monitoring of drug utilization to generate information for ensuring effective use of anti-malarial drugs in Nigeria. This information is currently limited in the retail sector which constitutes a major source of malaria treatment in Nigeria, but are characterized by significant inappropriate use of drugs. This study analyzed the use pattern of anti-malarial drugs in medicine outlets to assess the current state of compliance to policy on the use of artemisinin-based combination therapy (ACT).

**Methods:**

A prospective cross-sectional survey of randomly selected medicine outlets in Enugu urban, southeast Nigeria, was conducted between May and August 2013, to determine the types, range, prices, and use pattern of anti-malarial drugs dispensed from pharmacies and patent medicine vendors (PMVs). Data were collected and analyzed for anti-malarial drugs dispensed for self-medication to patients, treatment by retail outlets and prescription from hospitals.

**Results:**

A total of 1,321 anti-malarial drugs prescriptions were analyzed. ACT accounted for 72.7%, while monotherapy was 27.3%. Affordable Medicines Facility-malaria (AMFm) drugs contributed 33.9% (326/961) of ACT. Artemether-lumefantrine (AL), 668 (50.6%) was the most used anti-malarial drug, followed by monotherapy sulphadoxine-pyrimethamine (SP), 248 (18.8%). Median cost of ACT at $2.91 ($0.65-7.42) per dose, is about three times the median cost of monotherapy, $0.97 ($0.19-13.55). Total cost of medication (including co-medications) with ACT averaged $3.64 (95% CI; $3.53-3.75) per prescription, about twice the mean cost of treatment with monotherapy, $1.83 (95% CI; $1.57-2.1). Highest proportion 46.5% (614), of the anti-malarial drugs was dispensed to patients for self-treatment. Treatment by retail outlets accounted for 35.8% while 17.7% of the drugs were dispensed from hospital prescriptions. Self-medication, 82%, accounted for the highest source of monotherapy and a majority of prescriptions, 85.6%, was adults.

**Conclusion:**

Findings suggest vastly improved use of ACT in the retail sector after eight years of policy change, with significant contributions from AMFm drugs. However the use of monotherapy, particularly through self-medication remains significant with increasing risk of undermining treatment policy, suggesting additional measures to directly target consumers and providers in the sector for improved use of anti-malarial drugs in Nigeria.

## Background

Although malaria treatment policies are well established, with countries in Africa adopting artemisinin-based combination therapy (ACT) as first-line treatment for uncomplicated malaria, problems on implementation in many settings still persist, undermining the goals of malaria treatment policy [1,2]. Understanding the extent of these problems is essential for generating evidence for policy interventions to improve implementation. In Nigeria, although ACT has been adopted for first-line treatment of uncomplicated malaria since 2005, evidence abounds on the improper use of anti-malarial drugs, such as the use of monotherapy and other less effective anti-malarial drugs, as well as inappropriate use of ACT [2]. This is especially so in the retail sector where studies have reported significant inappropriate use of anti-malarial drugs [3-6]. Since the introduction of ACT in many countries, reports have shown that while public sector malaria treatment has largely conformed to policy recommendations, the private sector is significantly characterized by inappropriate use of anti-malarial drugs [3-7]. The use of monotherapy, inadequate use of ACT, fake and adulterated drugs is widely reported, increasing the risk of treatment failures and development of drug resistance. Reports indicated limited access to the new agents in spite of a wide range of anti-malarial drugs in circulation. Since introduction, ACT remains the most expensive anti-malarial agent compared to commonly used monotherapy, with a median cost of between US$5 and $11per adult dose [8,9]. In recognition of the role played by the private sector and the drug supply chain on the high cost of anti-malarial drugs, the Global Fund for HIV/AIDS, Tuberculosis and Malaria (GFATM), in collaboration with malaria partners, introduced the Affordable Medicine Facility-malaria (AMFm) in 2009, to reduce the cost of supply and improve access to the utilization of quality ACT in low-income countries [10]. This was complemented with public campaigns and targeted provider-training to increase uptake of effective anti-malarial drugs. Furthermore, the use of artemisinin monotherapy poses concern about the development of resistance of malaria parasites to artemisinin derivatives when not used in combination with partner drugs in line with recommendations [2,11]. With a variety of anti-malarial drugs in circulation in retail outlets, there are issues with drug quality and accuracy of dosing as a result of wide variations in brand formulations and composition of active ingredients [2]. Irrational provision and use of anti-malarial drugs constitutes a major risk of increasing *Plasmodium* resistance to effective products and treatment, undermining the goals of malaria control. Factors that contribute to inappropriate use of anti-malarial drugs are influenced by demand for drugs by consumers, such as costs, lack of information about appropriate treatment and difficulties in assessing quality treatment by patients [12]. Similarly, providers in the retail sector are often influenced by their knowledge, financial incentives, competition, perceptions of patients’ attitudes, and regulatory sanctions [12]. Okeke *et al*. went further to suggest that prescribing patterns are more likely to follow patient demands and expectations as well as profit motive rather than professional principles [3,5]. Adherence to anti-malarial treatment policy by providers and patients alike is essential to achieve the goals of the policy [4,13], and the retail sector as a major provider of malaria treatment is key in achieving the objectives. Since the greater number of malaria treatment services are provided through the private retail sector in Nigeria [14,15], as is the case in most other developing countries, the sector represents a greater risk of policy failure in view of significant inappropriate use of medicines. Appropriate attention to this sector is therefore critical to achieve the goals of malaria case management. Regular monitoring of drug utilization, as recommended by policy [16] becomes important when identifying opportunities for enhancing effective implementation of the ACT policy. This study aimed to analyse the current demand and utilization pattern of anti-malarial drugs in medicine retail outlets in Enugu urban, in relation to ACT policy in order to generate information for improving effective implementation of malaria treatment policy.

## Methods

### Study area and population

The study was conducted in the urban city of Enugu, capital of Enugu State, southeast Nigeria. The city is populated by 722,664 inhabitants according to the 2006 census. The population is predominantly Ibo ethnic group, who are mainly civil servants and businessmen, with a significant number of artisans. Of the 17 local government areas (LGAs) of the state, three make up the Enugu urban: Enugu East, Enugu South and Enugu North [2]. There are two tertiary health institutions, two secondary and about 15 primary health care facilities, as well as several private health care facilities, comprising private for-profit and private not-for-profit organizations. There are 236 medicine retail outlets, 75 pharmacies and 161 patent medicine vendors (PMVs). Retail pharmacies and PMVs are the two outlets licensed to sell and dispense drugs, including anti-malarial drugs. While pharmacies are licensed to dispense both prescription and over-the-counter (OTC) drugs, PMVs, operated by people who have no formal training, are licensed to sell only OTC medicines, even though they are known to deal with a wide range of drugs [3]. Similarly many pharmacies are either owned and/or manned by employees who received no formal/professional training. The study was undertaken in these two categories of retail outlets. Malaria is a major disease burden in the area with children and pregnant women the most vulnerable [17]. *Plasmodium falciparum* is the dominant malaria species, as in most of Nigeria and artemether-lumefantrine (AL) is first-line treatment for uncomplicated malaria since 2005. Shortly afterwards, artesunate-amodiaquine (AA) was added as an alternative first-line drug to AL. However, a wide range of ACT has been registered in the country for the first-line treatment of uncomplicated malaria, such as dihydroartemisinin-piperaquine (DHAPQ) and artesunate-mefloquine (ASMQ). In Enugu state there is a free maternal and child health (FMCH) programme, introduced by the government in 2008 to provide free medical treatment at public health facilities for children below five years and pregnant women. However, in spite of the existence of free treatment policy, many households still pay for malaria treatment as a result of frequent scarcity of anti-malarial drugs in government health facilities [18].

### Study design and data collection

A prospective, descriptive, cross-sectional survey was carried out in medicine retail outlets comprising pharmacies and PMVs, between the months of May and August, 2013. A pretested questionnaire of six key questions was designed to collect data on patients’ demographics, drugs demanded, drugs supplied, cost of prescription, co-prescribed medications, and mode of delivery. Data were collected on anti-malarial drugs sold to patients for self-medication (drugs specifically requested by a patient without formal prescription), recommendation by retail outlet, and by prescription from a hospital. In view of the challenges and sensitive nature of data collection from the retail outlets and nature of the study, convenient sampling approach was used to purposively select a representative number of outlets across seven sections of the Enugu urban city; it was not possible to conduct a probability sampling using a sampling frame. However the sampling was designed to include outlet type and utilization levels, such that outlets with extremely low utilization were excluded. Utilization was defined as the rate of sales of anti-malarial drugs. Outlets with fewer than 20 anti-malarial drugs per week were not considered, as they would not be adequate to capture the pattern of drug-use compared to those with higher rates. The outlets were selected to cover all parts of the city and each outlet type (pharmacy and PMV), informed by estimated number of retail outlets in a section and expected sales of anti-malarial drugs. 20 outlets that agreed to participate and met criteria were initially selected during the selection period but four were dropped due to incomplete and inconsistent data on age, gender, anti-malarial drug and concomitant medications dispensed. Accordingly, at least two outlets (one pharmacy and one PMV) were selected from each area, and a total of 16 outlets were selected.

Initial visits were carried out to the outlets to discuss the study, obtain permission and agree on date of survey. During the period, investigators observed the outlets’ routine drug dispensing and documentation processes, examined prescriptions for necessary information and updated outlet staff on collection of appropriate information from clients or potential patients. Trained research assistants were engaged to participate in data collection by assisting outlet staff in collecting relevant information. Data were collected over ten consecutive days from each outlet over the three-month period.

For the purposes of the study, the drugs dispensed were categorized into three groups: those dispensed by prescriptions from hospitals or health facilities, prescription by the outlets (when treatment is provided by outlet), and for self-treatment by patients (when a patient specifically requests and purchases a particular anti-malarial drug, which is taken to mean treatment that does not involve consulting a health care provider). If a parent requested for and purchased a particular anti-malarial drug for his/her child from an outlet, this was considered self-treatment. However, the limitation of this definition of self-treatment is recognized, because some requests may have involved recommendations from friends and associates, which is comparable to consulting health centres [19]. It was necessary to use this definition because the demand pattern was measured by the number and type of drugs actually dispensed. It should be noted that when the same drug was available in different strengths, it was counted as one item. Similarly, if the same drug was available by different routes of administration, it was counted as one item. Combinations of drugs were also treated as a single item.

The drugs were identified and categorized as ACT and monotherapy based on current malaria treatment policy. Monotherapy was further divided into artesunate monotherapy and non-artesunate monotherapy. Their use was then analyzed by gender, age categories, mode/source of treatment, and outlet type. The range, prices and cost of treatment were similarly analyzed.

### Statistical analysis

The statistical software Statistical package for Social Science (SPSS) version 16 (SPSS Inc, Chicago, IL, USA), was used to analyze the data. Quantitative variables were described using appropriate summary statistics (mean, median, standard deviation, and range); categorical variables are presented using frequency and proportions. Association between two categorical variables was assessed using Chi-square test of independence. Data were analyzed at 5% significant level. Values of p < 0.05 were considered statistically significant.

### Ethical consideration

The study received approval from the Nnamdi Azikiwe University Teaching Hospital (NAUTH) Institutional Ethical Board, as part of a larger study on the pharmaco-economics of malaria treatment in Nigeria. An informed consent was obtained from staff at each outlet before commencing the study. Where necessary a patient consent was obtained before obtaining any information.

## Results

A total of 1,321 prescriptions were analyzed. Table 1 shows that there were more male encounters (55.9%) than females (44.1%). The majority of cases were adult prescriptions (85.2%), while children below the age of five years accounted for the least number of prescriptions (6.4%). ACT was received by 961 (72.7%) patients, while monotherapy was dispensed to 27.3%. AMFm drugs accounted for 23.5% (326) of anti-malarial drugs, representing 33.9% of ACT. Within the monotherapy category, artemisinin monotherapy was used in 17.8% of cases while sulphadoxine-pyrimethamine (SP) accounted for the highest proportion of non-artemisinin monotherapy dispensed to 68.9% of the group. Self-medication was responsible for the highest proportion of drug prescriptions (46.1%), while prescriptions from health facilities accounted for 18.2% of sample study.

**Table 1:** Demographic characteristics and summary findings

**SN**	**Variable**	**Number**	**Percentage (%)**
**1**	**Gender**		
	Male	738	55.9
	Female	583	44.1
**2**	**Age (years)**		
	Below 5	74	6.4
	5 to 12	113	8.4
	13 and above	1,134	85.2
**3**	**Drug category**		
	ACT	961	72.7
	Monotherapy	360	27.3
	Artemisinin monotherapy	64	17.8
	Non-artemisinin monotherapy	296	82.2
	AMFm	326	33.9
**4**	**Treatment mode**		
	Self-treatment	614	46.1
	Outlet treatment	473	35.7
	Prescriptions	234	18.2
**5**	**Average number of brands per outlet (standard deviation)**	18 (±4.47)	

ACT = artemisinin combination therapy; PMV = patent medicine vendor; AMFm = Affordable Medicine Facility–malaria.

### Range of anti-malarial drugs

**Figure 1:**
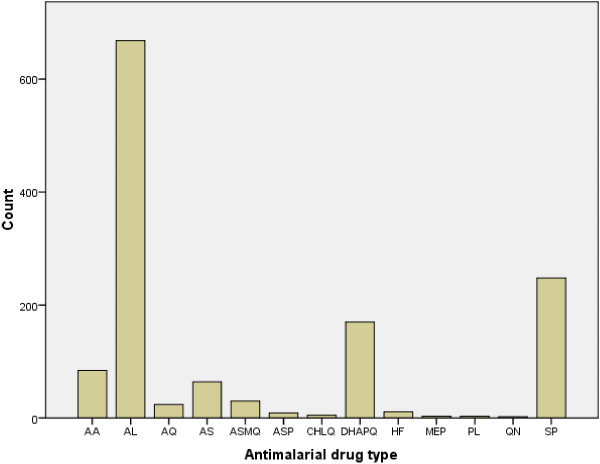
**The utilization pattern of anti-malarial drugs dispensed, by types.** AA = artesunate-amodiaquine; AL = artemether-lumefantrine; AQ = amodiaquine; AS = artesunate; ASMF = artesunate-mefloquine; ASSP = artesunate-sulphadoxine + pyrimethamine; CHLQ = chloroquine; DHAPQ = dihydroartemisin-piperaquine; HF = halofantrine; MEP = mepacrine; PL = proguanil; QN = quinine; SP = sulphadoxine + pyrimethamine.

A wide range of anti-malarial drugs was identified in a total of 13 different regimens, comprising over 75 brands of products, including tablets, suspensions and injections. Each outlet stocked an average of 18 (±4.47) brands. The number and frequency of use of the different types and combinations of anti-malarial drug regimens are shown in Figure 1. AL brands were the single most used regimen at 50.6% (668), followed by SP and DHAPQ at 18.8 and 12.9%, respectively. Chloroquine (CQ), proguanil (PL) and mepacrine (MEP) were the least used agents, as shown in Figure 1. ACT was available in six different combinations or regimens, accounting for the widest range of anti-malarial drugs dispensed. AL brands made up the highest number of brands within the entire anti-malarial regimens. AMFm drugs were found in every outlet surveyed.

### Utilization pattern

Table 2 shows the distribution of anti-malarial drugs by category, their mode of treatment and the pattern of use. Pattern of use was measured by the proportion of anti-malarial drug-use between ACT and monotherapy in each category. Females were more likely to use ACT (74.3%) than males (71.3%), although this was not statistically significant (p >0.05). Across the age groups, children were more likely than adults to use ACT (OR = 3.81; p < 0.0001). Use of ACT under the treatment modes shows that the highest proportion of use occurred among hospital and outlet prescriptions, at 91% (218/204) and 90% (425/472), respectively, while monotherapy was used most under self-medication by patients (47.4%). In retail outlets, there was little difference between the pharmacies, (74%) and the PMVs (70.7%) in the use of ACT, which was not statistically significant (p > 0.05). As shown in Table 2, the highest proportion of AMFm drugs (54/102) as ACT was used in children aged five to 12 years. In adults over 12 years old, AMFm represented only 31% of ACT dispensed.

**Table 2:** Distribution of anti-malarial drugs and mode of treatment across demographic groups and sources of treatment

	**Gender**		**Age group**			**Mode of treatment**			**Outlet type**	
	**Male**	**Female**	**<5 years**	**5-12 years**	**≥13 years**	**Self-treatment**	**Outlet treatment**	**Prescription**	**Pharmacy**	**PMV**
	**n (%)**	**n (%)**	**n (%)**	**n (%)**	**n (%)**	**n (%)**	**n (%)**	**n (%)**	**n (%)**	**n (%)**
Drug category										
ACT	527 (71.4)	434 (74.3)	74 (87.1)	102 (91.9)	785 (70.0)	318 (52.2)	425 (90.)	218 (90.8)	594 (74.1)	367 (70.7)
Monotherapy	211 (28.6)	149 (25.6)	11 (12.9)	9 (8.1)	340 (30)	291 (48.8)	47 (10.0)	22 (9.2)	208 (25.9)	152 (29.5)
Total	738	583	85	111	1125	609	472	240	802	519
Treatment mode										
Prescription	132 (17.9)	108 (18.5)	34 (40.0)	25 (22.5)	181 (16.1)	-	-	-	138 (17.2)	102 (19.6)
Recommendation	263 (35.6)	209 (35.9)	31 (36.5)	44 (39.6)	397 (35.3)	-	-	-	262 (32.7)	210 (40.5)
Self-treatment	343 (46.5)	266 (45.6)	20 (23.5)	42 (37.9)	547 (48.6)	-	-	-	402 (50.1)	207 (39.9)
Total	738	583	85	111	1125				802	519
AMFm	170 (32.2)	156 (36.0)	30 (40.5)	54 (53)	242 (31)	100 (32)	165 (39)	61 (28)	164 (27.6)	162 (44)

ACT = Artemisinin Combination Therapy; PMV = Patent Medicine Vendor.

Table 2 also shows that hospital prescriptions were the highest source of anti-malarial treatment for children aged below five years, at 40% compared to other modes, followed by outlet prescriptions. Only 16% of adult doses were dispensed from hospital prescriptions.

Study identified five different combinations or regimens of ACT and analysis showed that the AL brand was the most used ACT given to 70% (961) of the group. This was followed by DHAPQ (17.7%) and AA (8.7%) regimens, respectively.

The AMFm drugs were used most through outlet prescriptions where they accounted for 39% (165) of ACT dispensed under this mode. They represented 44% (162) of ACT dispensed through PMVs, and 27.6% through pharmacies.

### Co-prescribed medication

Some 70% (919) of dispensed drugs contained at least one concomitant medication. Some 51.7% (671/1,321) of patients received analgesics as the most commonly used concomitant medication, followed by antibiotics given to 18% (248/1,321) of the patients. Vitamin preparations were contained in 16.5% (218) of co-prescribed medications. Up to five drugs were used per patient at a median of one drug per patient. The majority of hospital prescriptions, 85% (195/240), contained concomitant medications while the least co-medications were observed for children between five and 12 years old, 54% (60/111).

### Drug prices and costs of medication

Table 3 presents the median prices of anti-malarial drugs as well as the mean/median cost of treatment including co-medication. Altogether, anti-malarial drug prices ranged from $0.19-13.55, at a median cost of $2.26 per dose. ACT was the most expensive with a median cost of $2.9 per dose ($0.65-7.42). This translates to three times the median cost of monotherapy at $0.97($0.19-13.55). AMFm drugs cost between $0.65 and 2.58 at a median price $1.94 per dose. Differences in the median prices and costs of treatment are reflected across the various groups and categories. While the lowest median price of $1.61 per anti-malarial drug and lowest treatment cost of $2.06 was recorded for children between five and 12 years, the highest price and cost of treatment was estimated for the hospital prescription mode at $3.16 and $3.61, respectively.

**Table 3:** The median prices and drug treatment costs of anti-malarial drugs in the surveyed retail outlets, across categories

	**Drug price (Naira)**		**Treatment cost (Naira)**			
**Variable**	**Median**	**Range**	**Median**	**Range**	**Mean**	**95% CI**
**Anti-malarial drugs**	350	30-2,100	420	50-4,800	496.77	478.03-515.51
ACT	450	100-1,150	500	100-2,600	575.78	557.48-594.08
Monotherapy	150	30-2,100	175	50-4,800	285.86	244.60-327.12
AMFm	300	100-450	345	100-1,700	349.82	335.82-363.81
**Gender**						
Male	350	50/1,550	420	60–2,670	500.49	476.56-524.42
Female	350	30/2,100	410	50-4,800	492.07	462.23-521.91
**Age group**						
Under 5	300	100/1,250	450	100-2,250	556.24	470.49-641.98
5-12 yrs	250	70/900	320	70-1,400	397.39	351.83- 442.95
over 12	350	30/2,100	420	50-4,800	502.08	481.59-522.58
**Treatment mode**						
Prescription	490	70/2,100	560	90-4,800	678.25	619.73-736.77
Outlet treatment	350	60/1,900	450	70-1,900	508.99	485.86-532.14
Self-medication	300	30/1,550	320	50-2,670	415.78	389.62-532.14
**Outlet type**						
Pharmacy	350	30/2,100	450	50-4,800	548.61	521.61- 575.61
Patent medicine vendor	300	70/1,500	380	70-1,650	416.67	395.20-438.14

ACT = artemisinin-based combination therapy; AMFm = Affordable Medicine Facility-malaria; US$1 = N155.

The total cost of medication (including co-medications) with ACT averaged $3.64 (95% CI; $3.53-3.75) per case, which is about twice the mean cost of treatment with monotherapy, at $1.83 (95% CI; $1.57-2.1). The average medication cost differed remarkably across age categories. While it was highest for children under five years at $3.32 (95% CI; $2.76-3.86), the lowest was observed for children aged between five and 12 years at $2.44 (95% CI; $2.16-2.72). Total medication cost also showed significant differences across treatment modes, with hospital prescription having the highest cost at $4.18 (95% CI; $3.81-4.55). Self-treatment had the least medication cost at $2.66 (95% CI; $2.50-2.83) per case.

## Discussion

This study, unlike previous studies, relied on actual drug utilization data to assess implementation of malaria treatment policy in the retail sector. Compared to previous studies [2], findings suggest vastly improved use of ACT in the sector, some eight years after the introduction as the first-line treatment for uncomplicated malaria in Nigeria. The study by Mangham *et al.* in 2009, about five years after policy change in the same area suggested utilization rate of 24.2% for ACT in medicine outlets using patients’ exit questionnaires [2]. Findings corroborate the results of previous, related studies in the area in terms of availability and utilization of anti-malarial drugs [2,5,6,8]. Prescriptions were mostly adults, while children cases were limited, comprising prescriptions from hospitals. Predominance of adult prescriptions agrees with previous findings that adults make more use of retail outlets than children [1,7]. The study shows that AL, as the policy first-line drug in Nigeria, is the most commonly used ACT followed by DHAPQ, corroborating the studies in 2009 by Magham *et al*. [2] and in 2010 by Uzochukwu *et al*. [6], which documented similar findings for both public and private health facilities in the same area. Studies in Uganda and Zambia also suggested preference for AL regimen over other ACT in health facilities [20]. The use of AA brands in this study appears quite limited at 8.7% of ACT even though it was the alternative first-line drug in Nigeria. Safety concerns associated with the use of AA is the possible reason for this, in addition to availability of a wide range of other ACT. AA has been associated with varying degrees of side-effects, which have limited its use, especially in adult patients [21-23]. The preference for AL may reflect expectation of efficacy and/or safety in the study environment. Other likely factors include availability of formulations and promotional activities of pharmaceutical companies operating in the country. In consequence, the AL regimen had the widest range of brands in the study as well as the most available. The high proportion of dispensed ACT, therefore, indicates a positive development on the part of retail outlets in the provision of malaria treatment. This can be attributed to several interventions that have been implemented in the sector to enhance appropriate use of anti-malarial drugs. These include the introduction of AMFm drugs accompanied by public campaigns and targeted provider trainings to enhance uptake of effective anti-malarial drugs, as well as community-based interventions [2,10]. The appreciable presence of AMFm drugs, which accounted for up to 23% of dispensed anti-malarial drugs, reinforced the use of ACT based on affordable prices. Consequently, their use impacted positively on prices and costs of treatment.

The study indicates that monotherapy, either as artemisinin- or non-artemisinin-based monotherapy was used significantly at 27% in the retail sector. The use of artemisinin-based monotherapy is contrary to policy recommendation in view of potential risk of parasite resistance as a result of its short half-life [11]. The extent of monotherapy in this study suggests substantial inappropriate use of anti-malarial drugs, which undermines treatment goals by promoting development of drug resistance and treatment failures, and should be a cause for concern. Analysis suggests that monotherapy was used more in adults and in PMVs, and obtained mostly through self-medication. This points to the need for more public education for appropriate use of anti-malarial drugs and better-targeted strategy to improve the use of ACT through PMVs and pharmacy sales staff, since they are known to command a significant population seeking malaria treatment in Nigeria [4,15]. Reason for use may be attributed to the higher costs of ACT, availability of monotherapy agents and lack of adequate information on appropriate use of effective anti-malarial drugs. This situation is likely to be worse in rural areas where the level of awareness is lower and choice may be more limited [21].

The extent of monotherapy is reinforced by the prevalence of self-medication as the highest source of drug utilization in this study. This was not surprising because previous studies have documented widespread self-medication among patients with anti-malarial drugs obtained from retail outlets, in most developing countries [15,19,24]. It was therefore expected that self-medication was the highest source of monotherapy, due to lack of adequate knowledge of effective anti-malarial drugs on the part of consumers, leading to inappropriate use of drugs. Self-treatment may be attributed to previous experience with a particular drug, such as having used the same drug for similar symptoms, or neighbour, friend, or relative previously taking the same drug for similar symptoms [25]. Self-medication, which is mostly based on presumptive diagnosis, will similarly be faced with consequences of misdiagnosis, over-treatment of malaria, masking of underlying, potentially fatal conditions and unnecessary side-effects [26,27]. This reinforces the increasing calls for interventions to improve treatments from these outlets through regular education programmes [3,12,28].

Outlet prescriptions reflect malaria treatment practices in medicine outlets, in terms of the use of ACT and findings suggest significant use of ACT, especially in pharmacies. This indicates positive impact of educational programmes on effective malaria treatment implemented in the past, as part of intervention strategies to educate private providers. It is expected that regular training of these providers translates to appropriate use of anti-malarial drugs. However the extent of monotherapy use in this study, especially through the PMVs, suggests the need for reinforced and regular training and education for these providers on effective use of anti-malarial drugs. The little difference in the use of ACT between the pharmacies and the PMVs is notable and can be explained by the significant presence of sales staff who do not have formal training, and which is expected to impact on service delivery through pharmacies. The successes achieved so far with the training programmes point to the usefulness of targeted interventions. Evidence of improved performance of the providers through targeted training has been demonstrated in studies carried out in Kenya and Tanzania [28-30]. Prescriptions from hospitals which consists almost entirely of ACT is consistent with findings that the public sector conforms more to policy on the use of ACT and that prescribing pattern is not influenced by patient demand, compared to the private retail sector [3,5,6]. Fewer cases of monotherapy prescription through this mode may be explained by the use of SP for prophylaxis in pregnancy. This may have contributed to making SP the highest used monotherapy in this study.

A major issue of concern in malaria treatment through the retail sector is presumptive treatment. Studies have shown that treatment through the medicine outlets is mostly based on clinical symptoms and this has been shown to result to over 50% being non-malaria cases [6,15], leading to wastage and inappropriate management of fevers and other complications. Although data was not comprehensively collected on laboratory diagnosis in this study, evidence suggests that the majority of malaria treatment was not supported by laboratory diagnosis. This is reinforced by the fact that anti-malarial drugs are treated as OTC medicines and the use is not regulated as prescription, even though regulation of drug sales in Nigeria, as in most developing countries, is not enforced. Some patients undertake diagnostic tests for malaria in private laboratories before visiting the outlets for treatment, sometimes with prescriptions from laboratory attendants [31]. This study suggests a high level of presumptive malaria treatment, which encourages the use of wrong drugs, limiting the use of effective anti-malarial drugs. Patient demand for diagnostic testing was limited, in addition to limited accessibility of laboratory services. Policy should, as a matter of urgency, consider the introduction of rapid diagnostic tests (RDT) in retail outlets to enhance accuracy and efficiency of malaria treatment in the sector. The World Health Organization (WHO) current approach to effective malaria treatment emphasizes the ‘test, treat and track’ (TTT) strategy, which translates to malaria diagnosis, early treatment with effective anti-malarial drugs and follow-up monitoring to ensure effective implementation.

Even though this study may not have adequately captured the extent of co-medication in the study, analysis indicates a substantial degree of concomitant treatment with other drugs. Understandably, analgesics were the most frequently used, followed by antibiotics with implications for increased cost of treatment. Vitamin preparations were the third most used co-medication. Analgesics are often required to relieve accompanying fevers and pains in malaria infection even though many cases actually resolve with effective anti-malarial drugs. The use of vitamin preparations, especially those containing minerals and trace elements (such as zinc, iron (Fe2+), copper, etc) with anti-oxidant properties has implications for the effectiveness of ACT. Anti-oxidants, vitamin C and vitamin E have been found to interfere with artemisinin compounds, thereby reducing their efficacy [32,33]; the advice is they are used after the completion of ACT dose, if needed [34]. The extent of concomitant medication in this study indicates substantial wastage when viewed against the prevalence of presumptive treatment of malaria which, in addition increases inefficiency in malaria treatment. Co-medication with antibiotics was apparently based on presumptive co-morbidity with mostly typhoid fever, which is often informed by previous laboratory findings and experiences, while a few are informed by laboratory tests accompanying malaria treatment requests. Widespread co-medication with antibiotics has implications for safety in view of the potential for drug-drug interactions and other safety concerns. Antibiotics are known to have high incidence of adverse events, such as rashes and pruritus, which might even be attributed to the new anti-malarial drugs [21]. This study suggests that children aged below five years are more likely to be prescribed co-medication. The study in Ghana reported similar findings where children below five received more co-prescribed antibiotics than other age categories [21]. Co-morbidity with pneumonia and related conditions is commonly associated with childhood malaria, which informed earlier recommendation for symptomatic treatment of childhood fevers with a combination of anti-malarials and antibiotics [26]. The lower proportion of co-medication in this study compared to the Ghana study may be explained by the fact that the retail sector is characterized by self-medication, where the choice and number of drugs may be limited by cost of treatment. It is also very likely that this study did not capture all co-medications used among the cohorts, for some reasons. Many patients may still have other medications such as analgesics and multivitamins which are routinely used mostly in children. This is particularly so for prescriptions dispensed through self-medication. The use of antibiotic co-prescription would suggest less confidence of actual diagnosis of malaria, hence prescribing anti-malarials would be a ‘cover’ for potential infection or to prevent subclinical infection becoming manifest [21]. Similar findings were reported from a study in Tanzania for patients with a history of cough for which anti-malarials were prescribed [35]. The issue of concomitant medication also contributes to irrational use of anti-malarial drugs due to polypharmacy and increased cost of care. Just as in many other studies, over-use of medication in malaria treatment results in polypharmacy, encouraging poor adherence in addition to the risks of drug interactions, adverse drug reactions and in consequence, treatment failures.

Study findings suggest significant reductions in prices and costs of treatment based on previous studies. The study by Mangham *et al*. [2] on the treatment of uncomplicated malaria (five years after introduction of ACT) showed the median cost of adult dose of ACT in the retail outlets to be N600 (US$3.87). A previous study in 2010 showed a median price of the ACT to be N700 (US$4.52) per adult dose [8]. The current price of N400 (US$2.67) per adult dose therefore suggests a significant reduction from previous prices. This can easily be attributable to the penetration of AMFm drugs which showed wide utilization across ages and gender. However, at a median cost of $1.94 per adult dose, the current prices of AMFm drugs are still well above intended targets of US$0.42-1.00 to achieve expected goals [10]. Available information suggests limited availability of AMFm products below their market demand and as a result, are sold at costs higher than recommended prices, in response to market forces. The median cost of ACT at US$2.67 per adult dose remains high and unaffordable for many low-income patients who therefore resort to cheaper monotherapy, thus contributing to the incidence of monotherapy.

### Limitations

Although significant information was obtained from this study, there were limitations that need to be highlighted to better interpret and use the information. Although sampling may suggest bias towards outlets with high patient turnover, the pattern of utilization which cuts across different parts of the city reflects health-seeking behavior of patients using retail outlets and therefore likely represents sample population with malaria in the city [21]. The study did not collect further information to accurately analyze the use of monotherapy and determine those that were used appropriately for prophylaxis in pregnancy and children, as well as those purchased to complete a dose in single-dose combination. Similarly, information on laboratory diagnosis following anti-malarial drug treatment was not adequately collected due to inconsistency and this would have helped inform a more comprehensive analysis of the drug use pattern in the retail sector. Due to the challenges of data collection in the retail sector and their unwillingness to volunteer information, data may be incomplete and potentially unreliable; hence, the use of large data to enhance precision and the validity of findings, in addition to the use of trained field staff to assist outlet staff in data collection. In spite of these limitations study findings largely reflect the pattern of anti-malarial drug utilization in the retail sector.

## Conclusion

This study suggests a vastly improved and substantial uptake of ACT as the anti-malarial drug of choice for the first line treatment of uncomplicated malaria in medicine retail outlets in Nigeria, eight years after policy change. This portends positive development towards achieving the goals of malaria treatment, considering the challenges of the sector. Evidence suggests positive contributions from the AMFm drugs, which was accompanied by targeted education programmes to improve the use of effective anti-malarial drugs, as well as the availability of a wide range of ACT. However the use of monotherapy and ineffective drugs remain significantly high due mainly to the prevalence of self-medication as the predominant mode of malaria treatment in the sector, in addition to poor treatment practices of providers. This would certainly lead to increasing risk of development of parasite resistance to effective anti-malarial drugs and treatment failures, thereby undermining the goals of malaria treatment policy. In view of the challenges of the sector, characterized by self-medication and the presence of poorly informed providers, there is need for improved targeting of the general public and the retail providers for enhanced and sustained education on effective use of anti-malarial drugs. This is crucial if the goals of malaria treatment policy are to be achieved, considering the role of the sector in the provision of malaria treatment in Nigeria.

## Competing interests

The authors have declared that they have no competing interests.

## Authors’ contributions

CCE conceived and designed the study, collected and analysed data and wrote the manuscript. BOO participated in the study design, data collection, coordination and interpretation. OE, MO and COE assisted in study design, data interpretation and manuscript review. All authors read and approved final manuscript
